# Induced pluripotent stem cell-derived endothelial progenitor cells attenuate ischemic acute kidney injury and cardiac dysfunction

**DOI:** 10.1186/s13287-018-1092-x

**Published:** 2018-12-10

**Authors:** Wen-Ching Shen, Yu-Hsiang Chou, Hsiang-Po Huang, Jenn-Feng Sheen, Shih-Chieh Hung, Hsin-Fu Chen

**Affiliations:** 1 Drug Development Center, Institute of New Drug Development, Institute of Biomedical Sciences, China Medical University, Taichung, 404 Taiwan; 2 Graduate Institute of Physiology, National Taiwan University College of Medicine, Taipei, Taiwan; 3 Renal Division, Department of Internal Medicine, National Taiwan University Hospital, Taipei, Taiwan; 4 Renal Division, Department of Internal Medicine, National Taiwan University Hospital Jin-Shan Branch, New Taipei City, Taiwan; 5 Graduate Institute of Medical Genomics and Proteomics, National Taiwan University College of Medicine, Taipei, Taiwan; 6 Department of Pediatrics, National Taiwan University Hospital, Taipei, Taiwan; 7 Department of Biotechnology, National Formosa University, Yun-Lin, Taiwan; 8 Integrative Stem Cell Center, Department of Orthopaedics, China Medical University Hospital, Taichung, 404 Taiwan; 9 Institute of Biomedical Sciences, Academia Sinica, Taipei, 105 Taiwan; 10 Division of Reproductive Endocrinology and Infertility, Department of Obstetrics and Gynecology, National Taiwan University Hospital, Taipei, Taiwan

**Keywords:** Acute kidney injury, Indoxyl sulfate, Cardiac dysfunction, Endothelial progenitor cells, Induced pluripotent stem cells

## Abstract

**Background:**

Renal ischemia–reperfusion (I/R) injury is a major cause of acute kidney injury (AKI), which is associated with high morbidity and mortality. AKI is a serious and costly medical condition. Effective therapy for AKI is an unmet clinical need, and molecular mechanisms underlying the interactions between an injured kidney and distant organs remain unclear. Therefore, novel therapeutic strategies should be developed.

**Methods:**

We directed the differentiation of human induced pluripotent stem (iPS) cells into endothelial progenitor cells (iEPCs), which were then applied for treating mouse AKI. The mouse model of AKI was induced by I/R injury.

**Results:**

We discovered that intravenously infused iEPCs were recruited to the injured kidney, expressed the mature endothelial cell marker CD31, and replaced injured endothelial cells. Moreover, infused iEPCs produced abundant proangiogenic proteins, which entered into circulation. In AKI mice, blood urea nitrogen and plasma creatinine levels increased 2 days after I/R injury and reduced after the infusion of iEPCs. Tubular injury, cell apoptosis, and peritubular capillary rarefaction in injured kidneys were attenuated accordingly. In the AKI mice, iEPC therapy also ameliorated apoptosis of cardiomyocytes and cardiac dysfunction, as indicated by echocardiography. The therapy also ameliorated an increase in serum brain natriuretic peptide. Regarding the relevant mechanisms, indoxyl sulfate and interleukin-1β synergistically induced apoptosis of cardiomyocytes. Systemic iEPC therapy downregulated the proapoptotic protein caspase-3 and upregulated the anti-apoptotic protein Bcl-2 in the hearts of the AKI mice, possibly through the reduction of indoxyl sulfate and interleukin-1β.

**Conclusions:**

Therapy using human iPS cell-derived iEPCs provided a protective effect against ischemic AKI and remote cardiac dysfunction through the repair of endothelial cells and the attenuation of cardiomyocyte apoptosis.

**Electronic supplementary material:**

The online version of this article (10.1186/s13287-018-1092-x) contains supplementary material, which is available to authorized users.

## Background

Acute kidney injury (AKI) is a potentially devastating clinical problem [1]. Despite the availability of renal replacement therapy, AKI is associated with high mortality and morbidity [2–5]. When kidneys fail, dangerous levels of metabolites and waste products, including uremic toxins, accumulate in the body. Clinical evidence suggests that AKI is not only an indicator of illness severity but that it also leads to distant-organ injury and considerably affects mortality [6–10]. Grams et al. observed that AKI is not an isolated event and that it results in heart dysfunction through a proinflammatory mechanism involving inflammatory cytokine expression and increased oxidative stress [7]. A recent study further demonstrated that AKI may activate the production of dynamin-related protein 1 (Drp1) and may induce mitochondrial fragmentation in cardiomyocytes, thereby leading to cell apoptosis and cardiac dysfunction. Drp1 has thus become a new therapeutic target to alleviate AKI-induced cardiac dysfunction [10].

An increasing number of studies have provided evidence that cell therapy can lead to the repair of damaged kidney tissue; therapy with pluripotent stem cells has been demonstrated to lead to functional recovery in preclinical kidney models [11–13]. Induced pluripotent stem (iPS) cells can be obtained by reprogramming a broad range of adult somatic cell types to develop into embryonic stem cell-like pluripotent cells [14]. iPS cell technology represents a promising, novel strategy for the derivation of clinically applicable lineage-specific cells, such as endothelial progenitor cells (EPCs) [14–16]. Furthermore, iPS cells can be generated from cells from any part of an adult and exhibit potential for facilitating genetically matched “patient-specific” cell therapy, which would solve both ethical problems and immune system rejection [17, 18].

The enormous therapeutic potential of isolated human EPCs has been demonstrated for a wide range of ischemic tissues [19]. Many researchers believe that the therapeutic effect of these cells is mediated by their production of cytoprotective, anti-inflammatory, anti-apoptotic, and antifibrogenic factors as well as by their differentiation into specific cell types [20, 21]. Despite advances in adult stem cell technology, limited accessibility, limited numbers of functional cells, and cellular heterogeneity remain obstacles for drug discovery and successful application of regenerative medicine [13, 22, 23]. iPS cell therapy has led to functional recovery in animal models [24, 25]. However, therapy using iPS cells has also induced undesirable effects, including teratoma formation [13, 26]. Directing the differentiation of iPS cells into specific cell types for transplantation may be a more favorable option.

Yoo et al. induced the differentiation of human iPS cells into EPCs (iPS cell-derived EPCs, iEPCs) and confirmed the therapeutic effect of iEPC infusion in mouse models of hind limb ischemia and myocardial infarction [27]. Inspired by the aforementioned previous study, we considered whether therapy using iEPCs would result in protective effects in a mouse model of AKI induced by ischemia–reperfusion (I/R) injury. Our data revealed that iEPC therapy is a promising treatment strategy for AKI and remote cardiac dysfunction and that its protective effects are exerted through attenuation of apoptosis and inflammation.

## Methods

An expanded version of the “Methods” section is available in Additional file 1 and includes details on the following: approval for human studies, patients, generation of human induced pluripotent stem cells, characterization of iPS cell-derived EPCs, culture of HL-1 cardiomyocytes, tissue preparation for pathological examinations, scoring of tubular injury, evaluation of microvessels, identification of apoptosis by TUNEL staining, cell lysate preparation and Western blot analysis, evaluation of renal function, measurement of plasma interleukin-1β, measurement of plasma brain natriuretic peptide, measurement of plasma indoxyl sulfate, measurement of circulating human cytokines, and echocardiographic analysis.

### AKI model and iEPC therapy

Male (8–10 weeks) adult nonobese diabetic/severe combined immunodeficiency (NOD/SCID) mice were anesthetized using ketamine/xylazine (100/10 mg/kg, intraperitoneally) and were then subjected to right nephrectomy. Two weeks later, the left kidney was clamped for 24 min by using a nontraumatic micro-aneurysm clip (Karl Klappenecker, Tuttlingen-Nendingen, Germany) to induce an I/R injury under the homeothermic blanket system (Stoelting Co. Wood Dale, IL). In the blanket system, a rectal thermal probe and a heating pad were used to maintain the core body temperature at 37 °C. After removal of the clamp, blood flow reperfusion was confirmed visually. The mice were divided into two groups: AKI-iEPC mice and AKI-vehicle mice. In AKI-iEPC mice, 200 μL of phosphate-buffered saline (PBS) cell suspension containing 5 × 10^5^ iEPCs was infused via the tail vein 15 min after reperfusion. AKI-vehicle mice were infused with 200 μL of PBS. Sham controls underwent the same surgical procedure but without vascular occlusion. All of the animal experiments were performed in accordance with the details of the relevant guidelines and regulations and were approved by the Institutional Review Board of National Taiwan University Hospital.

### Statistical analysis

Data are expressed as mean ± standard error of the mean (SEM). The data were analyzed using GraphPad Prism (GraphPad Software, La Jolla, CA). One-way analysis of variance with post hoc analysis using Tukey’s method was conducted for multiple group comparisons. *P* < 0.05 was considered statistically significant.

## Results

### Increased plasma levels of creatinine, indoxyl sulfate, and interleukin-1β in patients with AKI

**Fig. 1:**
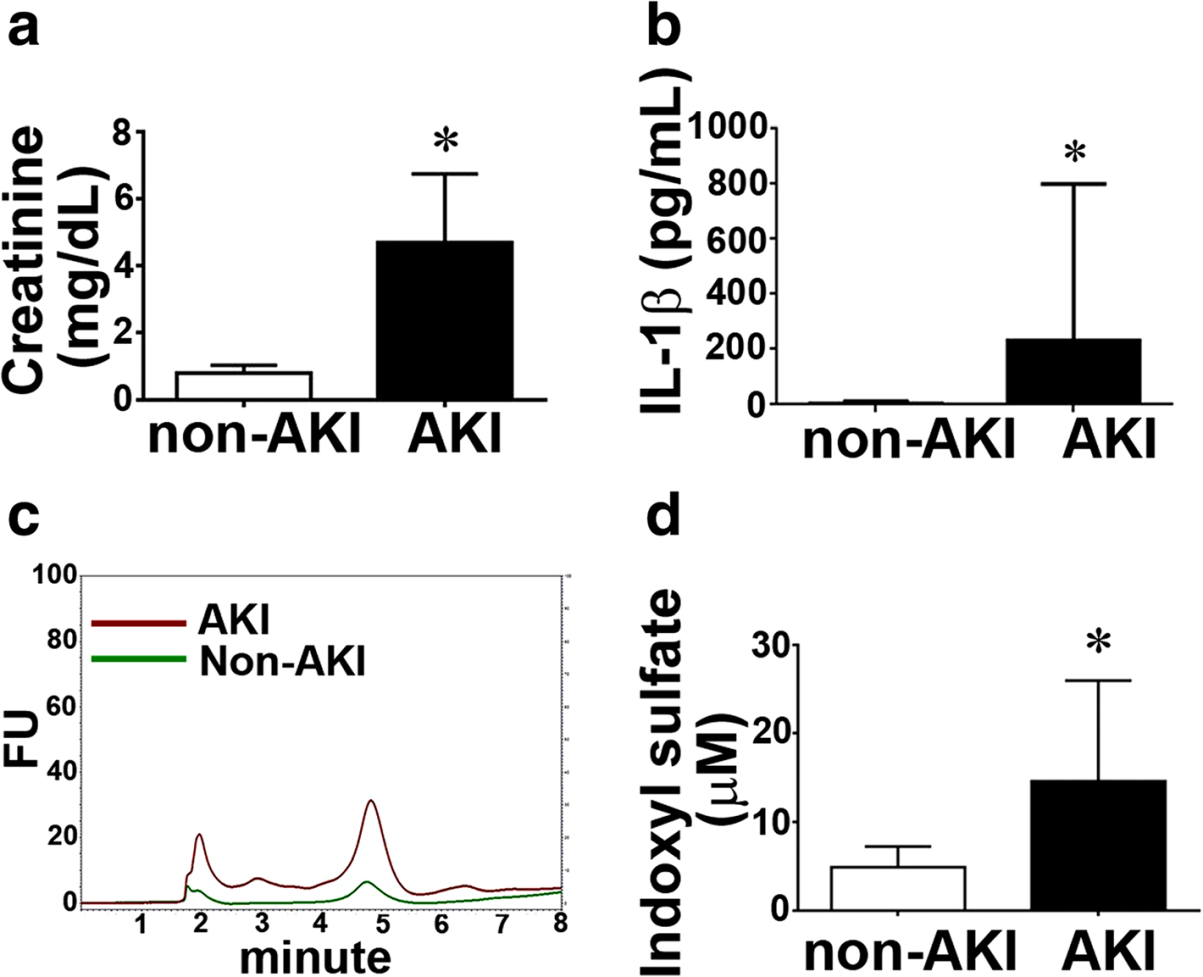
Increased plasma levels of creatinine and indoxyl sulfate in patients with AKI. **a** Bar chart denoting plasma creatinine levels in AKI and non-AKI patients. *N* = 25 per group. **b** Increased plasma levels of interleukin-1β in patients with AKI. Data are expressed as mean ± SEM. *N* = 10 per group. *P < 0.05 vs. non-AKI group. **c** Representative chromatogram for measurement of plasma indoxyl sulfate (IS). FU indicates fluorescence unit. **d** Bar chart representing plasma IS in AKI and non-AKI patients. Data are expressed as mean ± SEM. *N* = 6 per group. *P < 0.05 vs. non-AKI group

In the present study, 25 consecutive patients were enrolled upon receiving diagnosis of AKI. Day 2 serum level of creatinine was significantly higher in patients with AKI than in non-AKI controls (Fig. 1a). Serum levels of indoxyl sulfate (IS) and interleukin 1β (IL-1β) in each group were studied. Patients with AKI exhibited increased serum levels of IS (AKI, 18.1 ± 4.5 vs. non-AKI, 3.9 ± 0.6 μM; Fig. 1a, b) and IL-1β (AKI, 467 ± 50 vs. non-AKI, 97 ± 12 pg/mL; Additional file 1: Figure S2, Fig. 1c).

### Characterization of human iEPCs

A colony-forming unit of iEPCs was defined as a central core of round cells with elongated sprouting cells at the periphery (Additional file 1: Figure S1a and b). In contrast to iPS cells, iEPCs were spindle shaped in morphology when they reached confluence, similar to the morphology of mature endothelial cells (Additional file 1: Figure S1c and d). We also confirmed that acetylated low-density lipoprotein (acLDL) was taken up by iEPCs but not by iPS cells (Additional file 1: Figure S1e and f). In addition, primitive vascular tube-like structures developed in iEPCs when they were grown in Matrigel (Additional file 1: Figure S1 g and h). According to the results of immunostaining and flow cytometric analyses, both iPS cells and iEPCs expressed the stem cell marker CD133 (Additional file 1: Figure S2). However, only iEPCs expressed EPC marker kinase insert domain receptor (KDR; Additional file 1: Figure S2). CD31 and VE-cadherin, which are markers of mature endothelial cells, were also expressed in 57.6% and 53.3% of iEPCs, respectively (Additional file 1: Figure S2).

### Recruitment of iEPCs to the interstitium of AKI kidney

**Fig. 2:**
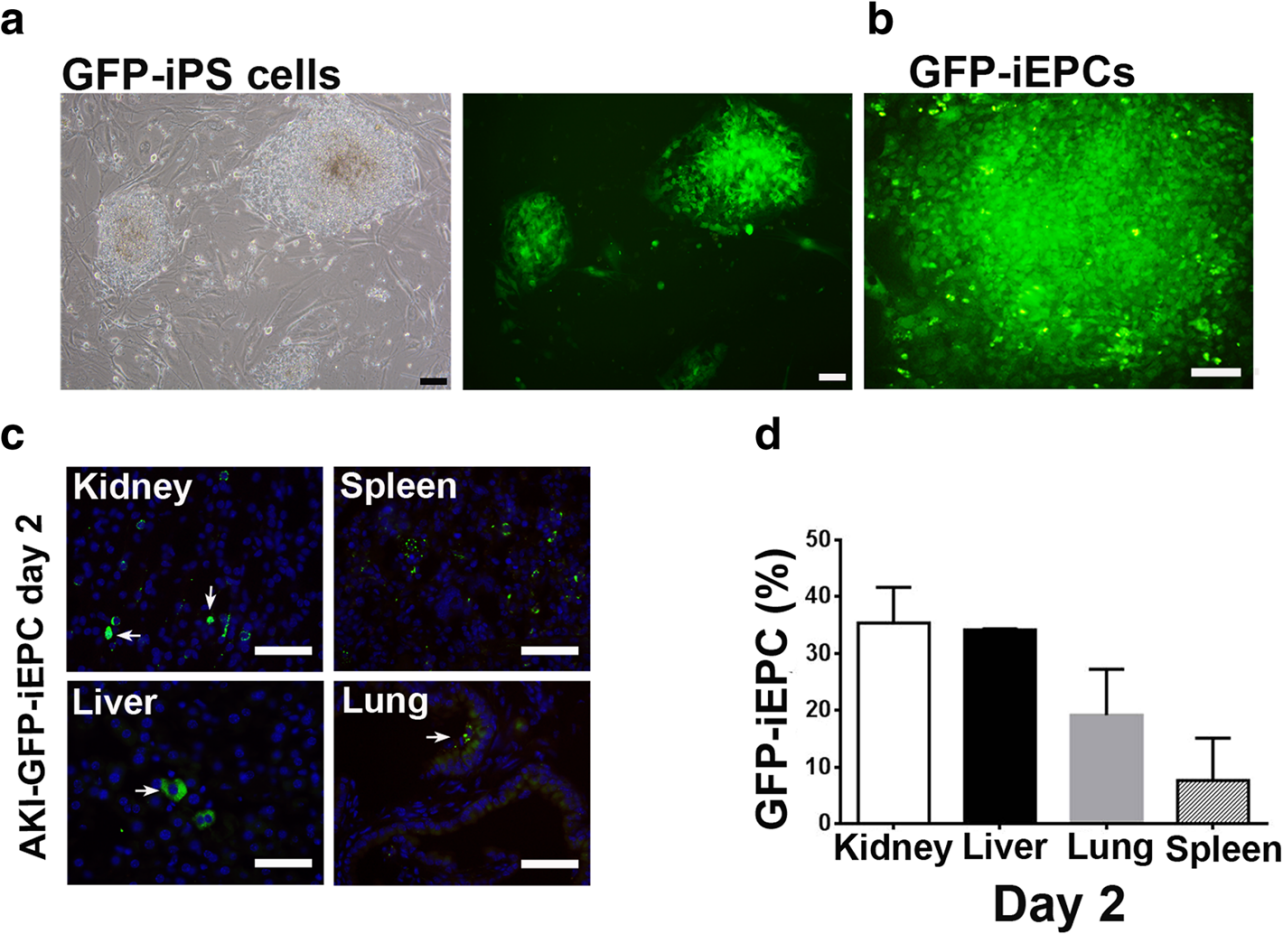
Intravenously infused human iPS cell-derived endothelial progenitor cells (iEPCs) were recruited to the post ischemia/reperfusion (I/R) injuries of the kidney, spleen, liver, and lung, but not the heart of nonobese diabetic/severe combined immunodeficiency (NOD/SCID) mice. **a** Bright-field and fluorescence images depicting undifferentiated colonies of lentivirus-GFP-transduced iPS cells on mitotically inactivated mouse embryonic fibroblasts. Scale bar = 100 μm. **b** Fluorescence images depicting a differentiated colony of GFP-iEPCs. Scale bar = 200 μm. **c** Fluorescence images depicting GFP-iEPCs recruited to the kidney, spleen, liver, and lung 48 h after intravenous infusion in mice with AKI induced by renal I/R injury. Arrows indicate GFP-iEPCs. Scale bar = 50 μm. **d** Bar chart denoting the percentage of recruited GFP-iEPCs per field captured at the original magnification × 400. The denominator is the sum of GFP-iEPCs in the kidney, spleen, liver, and lung. Data are presented as mean ± SEM. *N* = 4 per group

To assess the therapeutic effect of iEPCs on I/R-induced AKI, we first determined whether iEPCs could be recruited to the injured kidney. Lentivirus-green florescent protein (GFP)-transduced iPS cells were differentiated into GFP-iEPCs (Fig. 2a, b, Additional file 1: Fig. 3a, b). Fifteen minutes after reperfusion of ischemic kidneys, 5 × 10^5^ GFP-iEPCs were injected via the tail vein, and the kidneys were analyzed 2 days later (Fig. 2c, d). Many GFP-iEPCs were detected in the interstitium of the kidneys (Fig. 2c). As expected, GFP-iEPCs were also identified in the lung, liver, and spleen (Fig. 2c). However, among the organs examined, the proportion of GFP-iEPCs was highest in the kidneys (Fig. 2d). No GFP-iEPCs were observed in the heart.

**Fig. 3:**
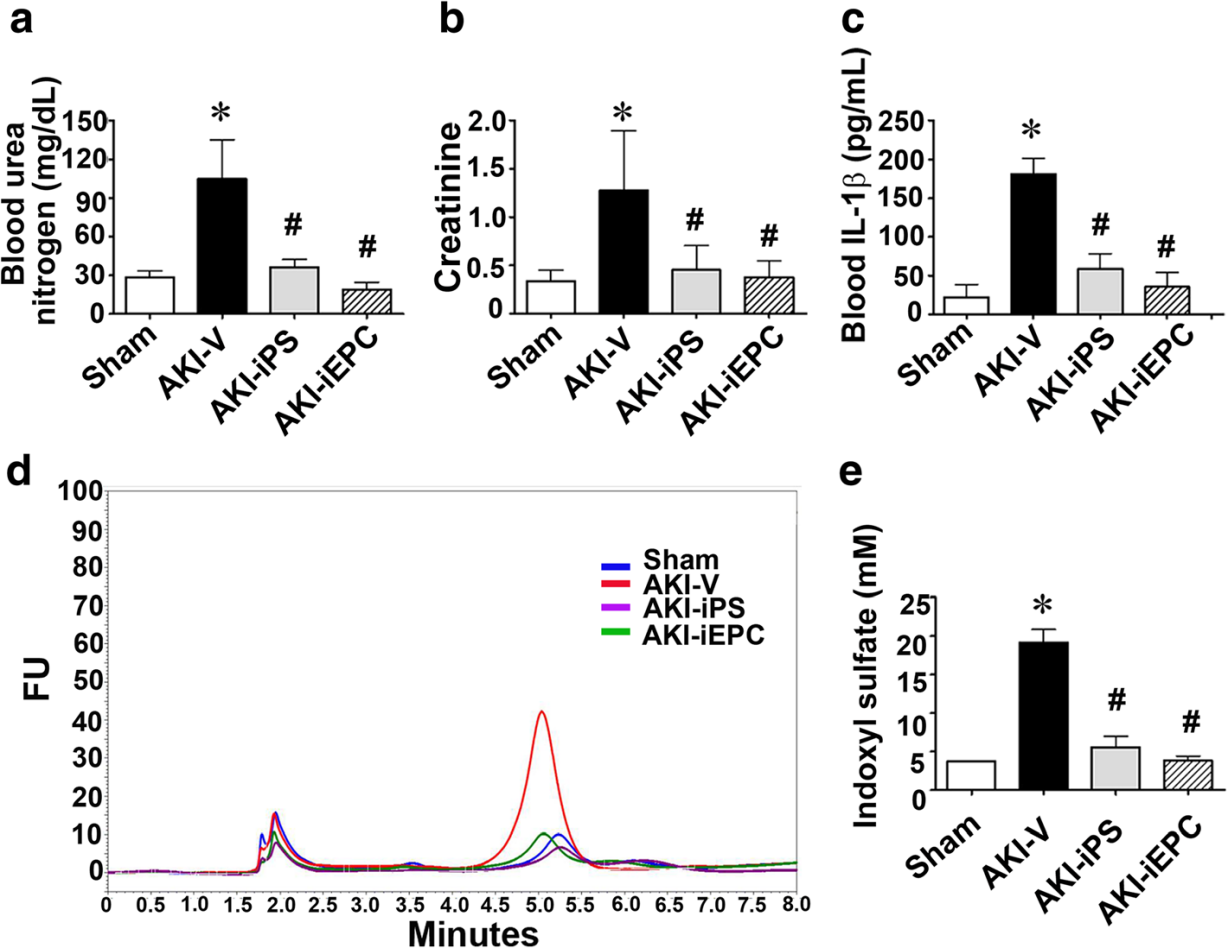
Systemic human iPS cell-derived iEPC therapy reduced azotemia and systemic inflammation after acute kidney injury (AKI). **a**–**c** Plasma levels of blood urea nitrogen (BUN) and creatinine and interleukin-1β (IL-1β) in mice subjected to sham surgery or renal I/R injury treated with vehicle (AKI-V) and systemic iEPC or iPS therapy (AKI-iEPC or AKI-iPS). *N* = 7–18 per group. **d** Representative image illustrating the measurement of plasma indoxyl sulfate by liquid chromatography. FU indicates fluorescence unit. **e** Bar chart denoting plasma levels of indoxyl sulfate in each group. *N* = 5 per group. Data are expressed as mean ± SEM. *P < 0.05 AKI-V vs. Sham, ^#^*P* < 0.05 AKI-iEPC or AKI-iPS vs. AKI-V group

### Systemic iEPC therapy reduced azotemia and systemic inflammation after AKI

To assess the therapeutic effect of iEPCs on AKI, iEPCs or PBS vehicle was injected via the tail vein 15 min after renal I/R surgery. Systemic iPS and iEPC therapy resulted in a significant decrease in plasma levels of creatinine (0.4 ± 0.1 mg/dL) and blood urea nitrogen (BUN; 19.1 ± 2.7 mg/dL) compared with the levels in vehicle-treated mice (creatinine, 1.3 ± 0.3 mg/dL and BUN, 105 ± 15.1 mg/dL; Fig. 3a). Similar to findings reported for patients with AKI (Fig. 1), plasma levels of IL-1β and IS were higher in AKI-vehicle mice than those in sham mice on day 2 after injury (Fig. 3c–e). iPS or iEPC therapy reduced the increase in plasma IL-1β and IS levels in AKI mice, and this result was comparable to the therapies’ beneficial effect on kidney function (Fig. 3c–e).

### Systemic iEPC therapy attenuated tubular injury and peritubular capillary rarefaction after AKI

**Fig. 4:**
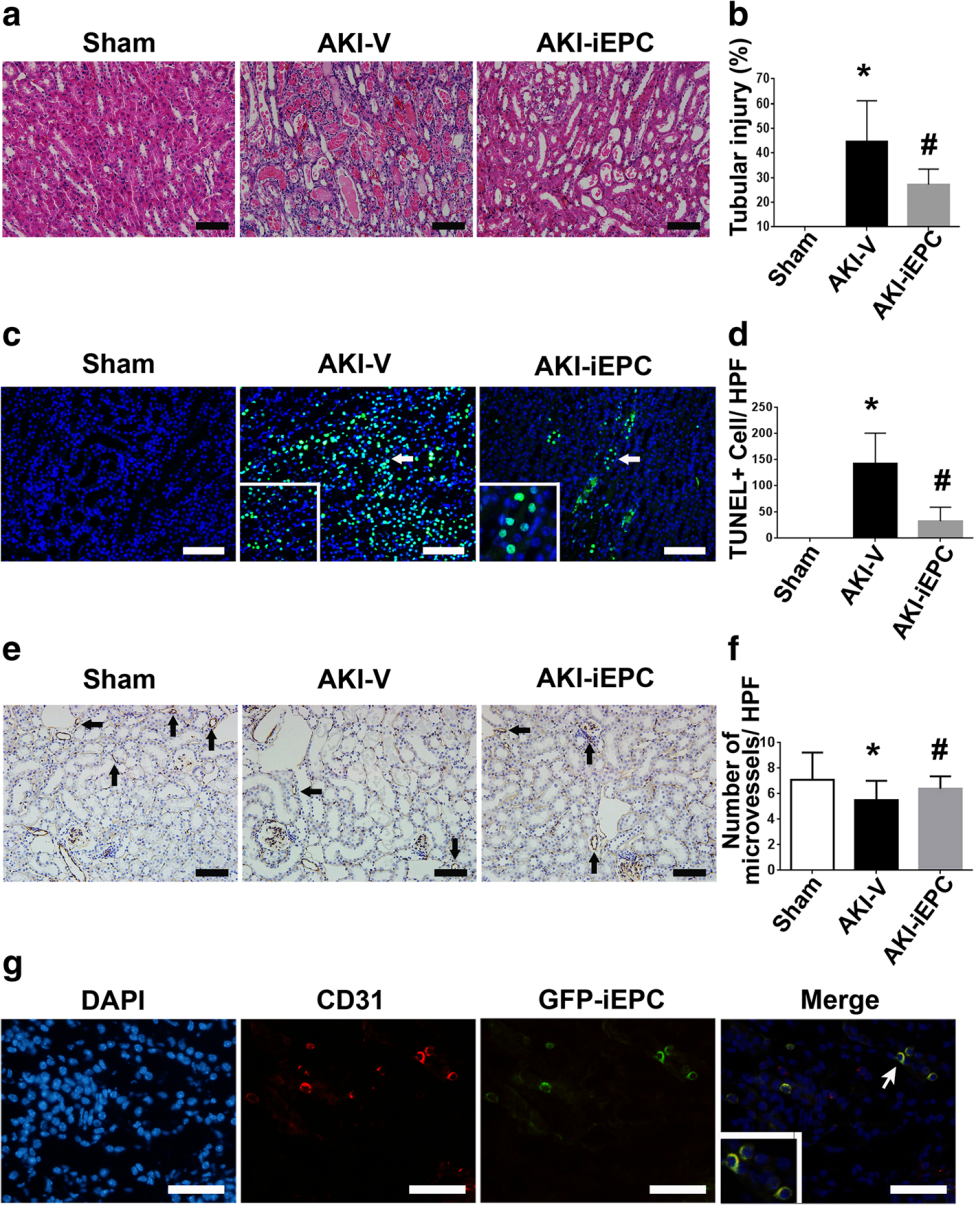
Systemic human iPS cell-derived iEPC therapy attenuated tubular injury and peritubular capillary rarefaction after acute kidney injury (AKI). **a** Representative hematoxylin and eosin-stained images depicting tubular injuries, including tubular necrosis, absence of nuclei, tubular dilatation, and intratubular cast, on day 2 after sham or AKI. **b** Bar chart representing semiquantitative analyses of tubular injury on day 2 after sham or AKI. *N* = 10 per group. **c** Representative immunofluorescence images of the detection of apoptosis through terminal dUTP nick-end labeling (TUNEL) staining on day 2 after sham or AKI. Arrows indicate TUNEL+ (green) nuclei (blue). **d** Bar chart representing quantitative analyses of the numbers of TUNEL+ apoptotic cells per high-power field (× 200 magnification) on day 2 after sham or AKI. *N* = 8 per group. **e** Representative immunohistochemical images depicting the staining of CD31+ endothelial cells on day 2 after sham or AKI. Arrows indicate CD31+ staining (brown). **f** Bar chart representing quantitative analyses of the number of microvessels per HPF on day 2 after sham or AKI. *N* = 10 per group. Data are expressed as mean ± SEM. *P < 0.05 AKI-V vs. Sham, ^#^*P* < 0.05 AKI-iEPC vs. AKI-V group. Scale bar = 100 μm. **g** Representative immunofluorescence images depicting recruited GFP-iEPCs expressing human CD31 on day 2 after AKI. Arrow indicates a GFP-iEPC expressing CD31. Scale bar = 50 μm

Given that iEPCs were recruited to the injured kidney and reduced azotemia, we next studied the mechanisms underlying the therapeutic effect. iEPC therapy induced restoration of the corticomedullary junction in the AKI-vehicle kidney on day 2 after I/R injury (Additional file 1: Figure S4). iEPC therapy also attenuated marked tubular injuries, including intratubular cast, absence of nuclei, and tubular dilation, in the AKI-vehicle kidney (Fig. 4a, b). Terminal deoxynucleotidyl transferase dUTP nick-end labeling (TUNEL+) apoptotic cells in the AKI-vehicle kidney were also attenuated by iEPC therapy (Fig. 4c, d). Decreases in CD31+ staining revealed rarefaction of peritubular capillaries in the AKI-vehicle kidneys, and this was also improved by iEPC therapy (Fig. 4e–f).

### Recruited iEPCs repaired endothelial cells in AKI kidneys and expressed proangiogenic factors

**Fig. 5:**
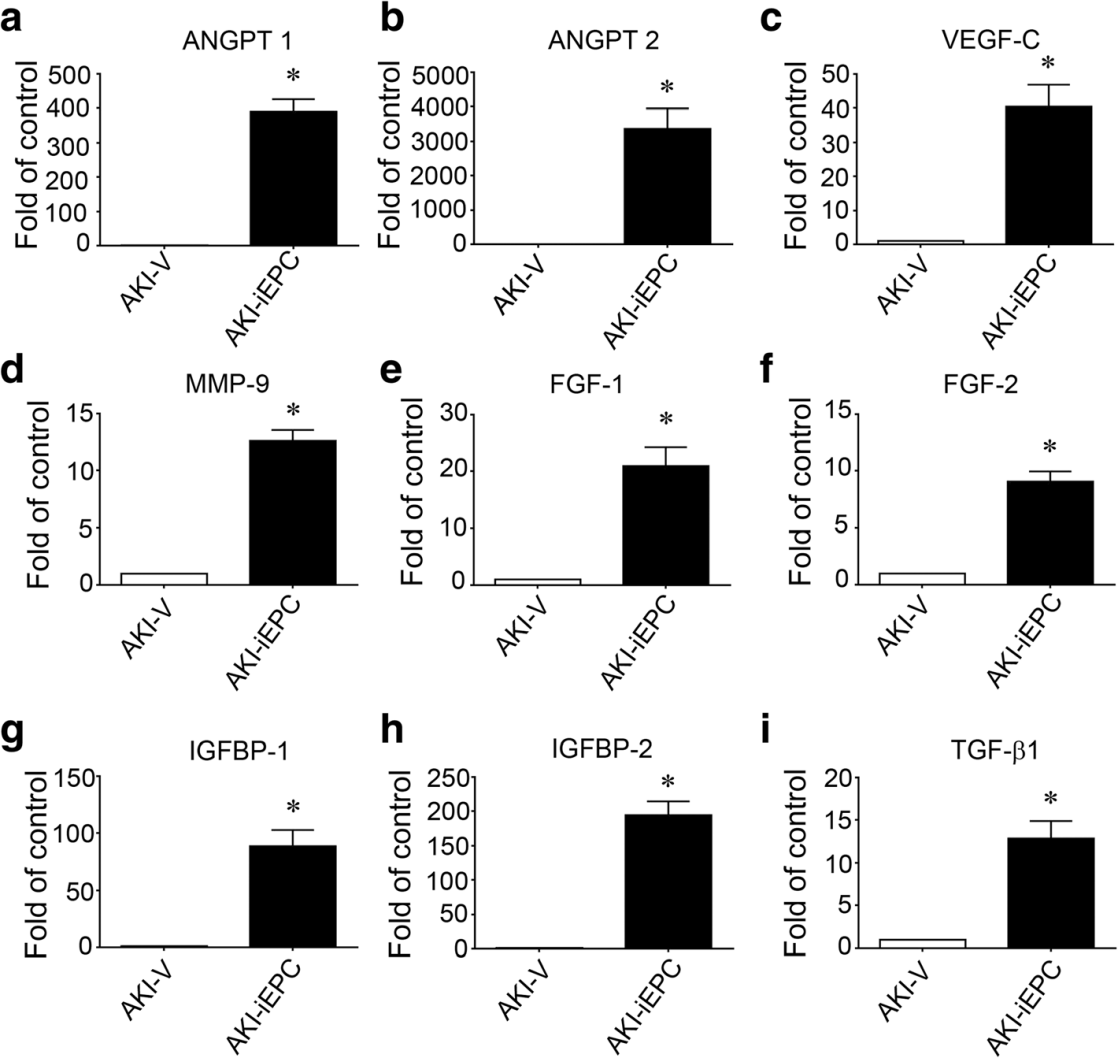
**a**–**i** Systemic human iPS cell-derived iEPC therapy increased the plasma concentrations of angiogenesis-related proteins. Bar charts denoting the relative folds of plasma angiogenesis-related proteins—including Angpt1, Angpt2, VEGF-C, MMP 9, FGF 1, FGF 2, IGFBP-1, IGFBP-2, and TGF-β1—on day 2 after acute kidney injury (AKI). *N* = 3 per group. Data are expressed as mean ± SEM. *P < 0.001 AKI-iEPC vs. AKI-V group

To further analyze the mechanism by which iEPCs maintained microvasculature in AKI kidneys, we determined whether iEPCs differentiated into endothelial cells. We found that all GFP-iEPCs were positive for human CD31 in the kidneys of AKI-iEPC mice, suggesting that iEPCs differentiated into mature endothelial cells (Fig. 4g). However, we discovered that GFP-iEPCs accounted for less than 1% of mouse CD31+ endothelial cells (data not shown). These investigations provided evidence of an extremely rare contribution of iEPCs to direct endothelial replacement, and the marked expression of the mature endothelial cell marker CD31 in all recruited iEPCs was noted; therefore, we measured circulating angiogenesis-related proteins to determine whether paracrine mechanisms underlie systemic-iEPC-therapy-induced maintenance of microvasculature in AKI kidneys. Compared with levels in AKI-vehicle mice, plasma levels of angiopoietin-1 (Angpt1), angiopoietin-2 (Angpt2), vascular endothelial cell growth factor-C (VEGF-C), matrix metallopeptidase 9 (MMP9), fibroblast growth factor-1 (FGF1), FGF2, insulin-like growth factor-binding protein-1 (IGFBP-1), IGFBP-2, and transforming growth factor-β1 (TGF-β1) had considerably increased in AKI-iEPC mice 2 days after injection (Fig. 5, Additional file 1: Figure S5).

### Systemic iEPC therapy attenuated cardiac dysfunction after AKI

**Fig. 6:**
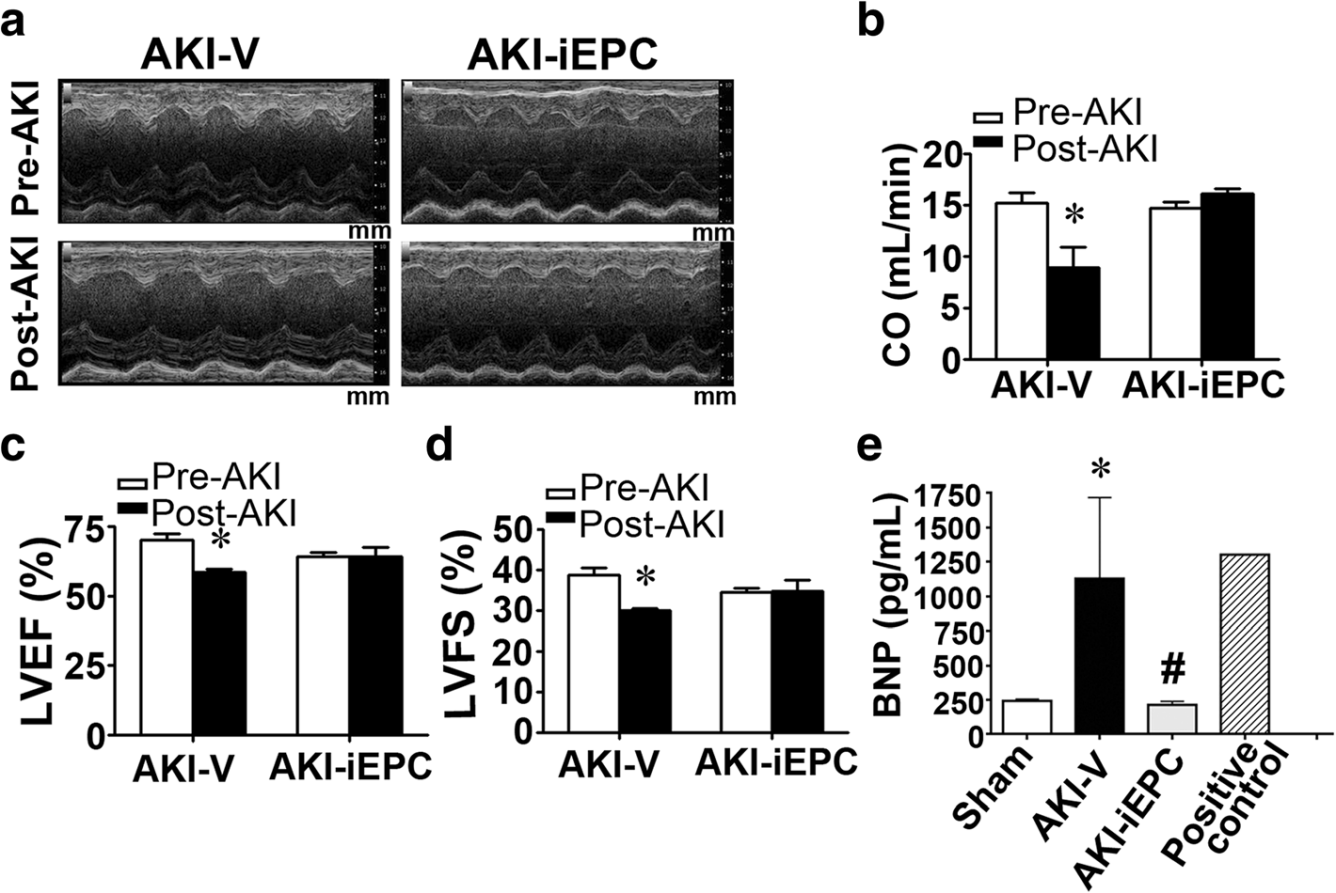
Systemic human iPS cell-derived iEPC therapy attenuated cardiac dysfunction after acute kidney injury (AKI). **a** Representative M-mode echocardiographic images of the left ventricles in mice before (Pre-AKI) or 2 days after AKI (Post-AKI). Mm (millimeter) indicates the depth of tissues. **b**–**d** Cardiac function parameters including cardiac output (CO), left ventricular ejection fraction (LVEF), and left ventricular fractional shortening (LVFS), as assessed using M-mode echocardiography. *N* = 6 per group. *P < 0.05 vs. Pre-AKI. **e** Plasma levels of brain natriuretic peptide (BNP). *N* = 5 per group. Data are expressed as mean ± SEM. *P < 0.05 AKI-V vs. Sham, ^#^*P* < 0.05 AKI-iEPC vs. AKI-V group

The plasma level of brain natriuretic peptide (BNP) was significantly higher in patients on day 2 after AKI, suggesting congestion of cardiac atria (Additional file 1: Figure S6). To assess cardiac function in AKI mice, echocardiography was performed on day 2 after I/R surgery. Representative M-mode tracings indicated significant improvement of cardiac function in AKI mice after iEPC therapy (Fig. 6a). Cardiac dysfunction—as evident by decreases in cardiac output (CO), left ventricular ejection fraction (LVEF), and left ventricular fractional shortening (LVFS)—in AKI mice was attenuated after iEPC therapy (Fig. 6b–d). In AKI mice, elevated plasma levels of BNP, which contributes to cardiac dysfunction, were substantially attenuated by iEPC therapy, and this result agreed with the findings from echocardiography (Fig. 6e).

### Systemic iEPC therapy attenuated apoptosis of cardiomyocytes in AKI mice

**Fig. 7:**
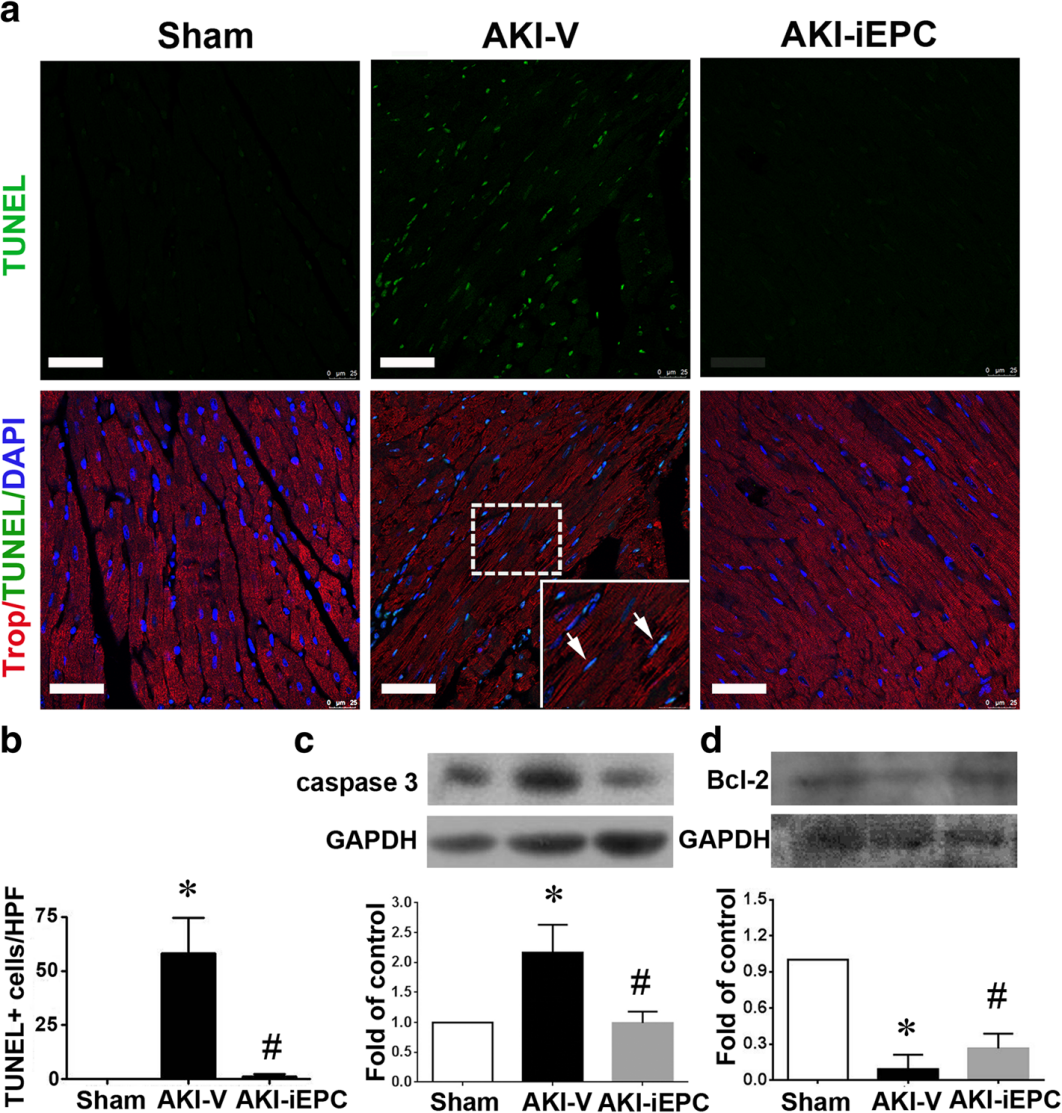
Systemic human iPS cell-derived iEPC therapy attenuated apoptosis of cardiomyocytes in acute kidney injury (AKI) mice. **a** Representative immunofluorescence images depicting TUNEL+ cells in the left ventricles on day 2 after sham or AKI. Arrows indicate TUNEL^+^ (green) nuclei (blue) and cardiomyocytes staining. Scale bar = 100 μm. **b** Bar chart illustrating quantitative analyses of TUNEL+ apoptotic cells per high-power field (× 200 magnification) in the left ventricle on day 2 after sham or AKI. *N* = 8 per group. **c**, **d** Representative images of Western blot analyses of caspase 3 and Bcl-2 in the left ventricles on day 2 after sham or AKI. GAPDH was used as the control of protein loading. Bar charts illustrating quantitative analyses. *N* = 3 per group. Data are presented as mean ± SEM. *P < 0.05 AKI-V vs. Sham, ^#^*P* < 0.05 AKI-iEPC vs. AKI-V group

Following the previously described experiments, we investigated the possible mechanisms underlying the protective effect of iEPC therapy on cardiac function in AKI mice. Apoptosis of left ventricular cardiomyocytes, which was assessed using terminal dUTP nick-end labeling (TUNEL) staining, was elevated in AKI mice (Fig. 7a, b), and iEPC therapy substantially reduced the elevated apoptosis (Fig. 7a, b). Moreover, the expression of the apoptotic protein caspase 3, which was elevated in the left ventricle of AKI mice, was also inhibited by iEPC therapy (Fig. 7c). By contrast, the downregulation of anti-apoptotic protein Bcl-2 in the left ventricle of AKI mice was partially attenuated by iEPC therapy (Fig. 7d).

### Indoxyl sulfate and interleukin-1β induced cardiomyocyte apoptosis synergistically

**Fig. 8:**
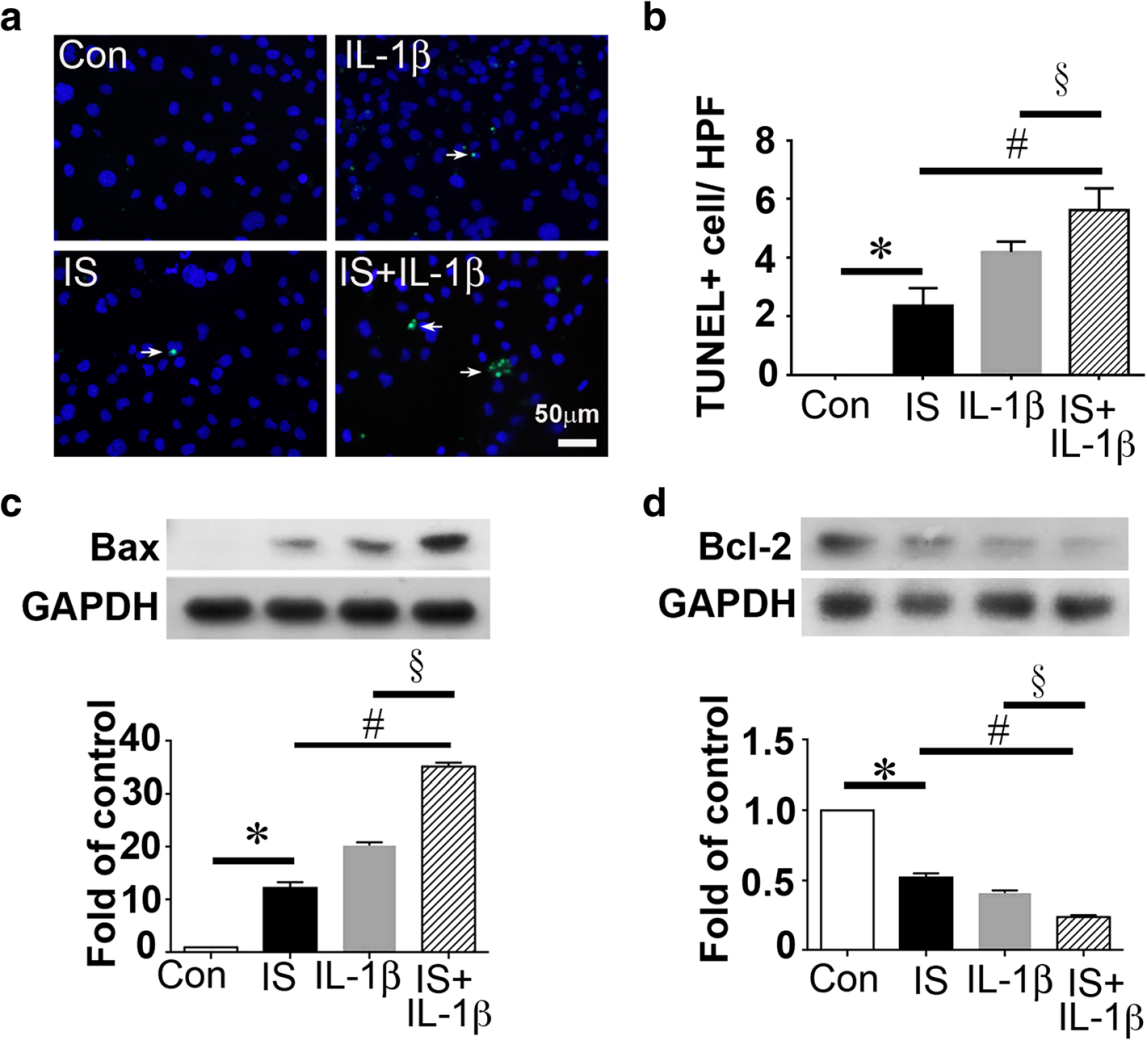
Indoxyl sulfate (IS) and interleukin (IL)-1β induced cardiomyocyte apoptosis after treatment with IS or IL-1β. **a** Representative immunofluorescence images presenting TUNEL+ (green) HL-1 cardiomyocytes cultured in medium with or without IS or IL-1β. Con indicates control. Scale bar = 50 μm. **b** Bar chart depicting quantitative analyses of TUNEL+ HL-1 cardiomyocytes per high-power field (× 400 magnification). **c**, **d** Representative images of Western blot analyses and the statistical analyses for Bax, Bcl-2, and GAPDH in HL-1 cardiomyocytes cultured in medium with or without IS or IL1-β. The protein levels of Bax and Bcl-2 were normalized using GAPDH in bar charts. *N* = 3 per group. Data are presented as mean ± SEM. *P < 0.05 IS vs. Con, ^#^*P* < 0.05 IL-1β + ΙS vs. IS, ^§^*P* < 0.05 IL-1β + ΙS vs. IL-1β

Based on the finding that IS and IL-1β increased systemically in AKI patients and mice (Fig. 1, Fig. 3c–e) and systemic iEPC therapy decreased IS and IL-1β in AKI mice, we determined whether IS or IL-1β played a role in the apoptosis of cardiomyocytes. At the concentrations (0.2 mM IS and 200 ng/mL) observed in patients with AKI, IS and IL-1β induced apoptosis of cardiomyocytes HL-1 (Fig. 8a, b). Moreover, IS and IL-1β synergistically induced cardiomyocyte apoptosis (Fig. 8a, b). Mechanistically, IS and IL-1β upregulated the proapoptotic protein Bax but downregulated the anti-apoptotic protein Bcl-2 in HL-1 cardiomyocytes (Fig. 8c, d).

## Discussion

Our findings provide the first evidence that human iPS cell-derived iEPC therapy is a promising therapy that may attenuate apoptosis and thereby protect the kidneys from microvascular rarefaction and tubular decomposition induced by I/R injury. The present study also revealed that AKI-induced cardiomyocyte apoptosis and cardiac dysfunction were attenuated by this therapy.

I/R injury is the most common cause of AKI in patients [1]. Vascular and tubular changes, alongside interstitial inflammation, cause acute decreases in kidney function. AKI-induced IS and IL-1β, the expression of which was examined in this study, damage endothelial cells and cause myocardial injury, respectively [28, 29]. Despite extensive research through preclinical studies, no therapeutic interventions using iPS cell-derived iEPCs have been reported to prevent or accelerate recovery from AKI [30]. This failure in translation has led investigators to speculate that the animal models and study designs used in the relevant research do not predict clinical responses. Our pilot experiments with mice subjected to bilateral renal I/R injury frequently resulted in unequal kidney sizes, with one atrophic kidney and another hypertrophic kidney 2 weeks after injury. These results demonstrated the technical difficulty of inducing equal injuries to both kidneys in a model of bilateral I/R injury. Therefore, in the present study, we performed right nephrectomy in 8–10-week-old mice, followed by left renal I/R injury 2 weeks later. This AKI model was reliable and facilitated the objective evaluation of kidney function and renal pathology. Our data revealed that intravenous infusion of iEPCs 15 min after renal I/R surgery provided protective effects not only for kidney function but also for distant organs such as the heart. Lentiviral transduction enabled reliable fate tracing of infused iEPCs, and the results indicated that GFP-iEPCs were mainly recruited to the interstitium of the injured kidney. Recruited iEPCs preferentially lined up with mouse CD31+ endothelial cells and upregulated their own CD31, indicating the potential of infused iEPCs to replace injured endothelial cells. This finding is in line with those of previous reports that demonstrated the homing of EPCs, isolated from various tissues, into injured blood vessels [31–40]. However, direct endothelial replacement by GFP-iEPCs only accounted for extremely small number of cells in blood vessels. Our data further supported that iEPC therapy might preserve microvasculature by causing the expression of high levels of angiogenesis-related proteins, including Angpt1, Angpt2, VEGF-C, MMP9, FGF1, FGF2, IGFBP-1, IGFBP-2, and TGF-β1. This finding was in line with evidence that EPCs release potent proangiogenic growth factors [41]. Proangiogenic growth factors appear to be produced not only by EPCs but also by hematopoietic stem/progenitor cells and bone-marrow-derived mesenchymal stem cells [11, 42]. In this study, we did not gain insight into the role of each angiogenesis-related protein expressed in AKI-iEPC mice. However, the proangiogenic properties of growth factors and effectors, including angiopoietin, CXCL8, and MMP9, have been demonstrated in previous studies involving neutralization of antibodies, specific antagonists, or genetic disruption against specific factors [43–47]. In addition, one independent study demonstrated that EPCs may exhibit cytoprotective effects through the broad synergistic action of many growth factors and effectors, resulting from modulation of intracellular anti-oxidative defensive mechanisms and prosurvival signals [48]. In this study, both direct endothelial replacement with iEPCs and iEPCs’ stimulation of the production of many proangiogenic growth factors and effectors were observed in AKI kidneys, and these effects contributed to the attenuation of microvascular rarefaction and tubular decomposition.

Our echocardiography data indicated that iEPC therapy exhibited a protective effect against AKI-induced cardiac dysfunction. We did not observe any GFP-iEPCs in mouse hearts, suggesting that the possibility of a direct cardiac effect was negligible. Considering that iEPC therapy prevented kidney injury, the protective effect of iEPC therapy on the heart may have resulted from the reduction of uremic toxin IS and proinflammatory IL-1β in circulation. Our study further supported that IS and IL-1β at the concentrations observed in patients with AKI were proapoptotic to cardiomyocytes. The number of apoptotic cardiomyocytes in AKI mice decreased after iEPC therapy, suggesting that iEPC therapy exerted prosurvival and anti-apoptotic effects resulting from the attenuation of AKI and the reduction of circulating IS and IL-1β levels. A recent study demonstrated that AKI may activate Drp1, inducing mitochondrial fragmentation and apoptosis in cardiomyocytes, but the mechanism underlying Drp1 activation and apoptosis in cardiomyocytes following renal I/R injury has not been identified [10]. In the present study, IS and IL-1β did not induce Drp1 activation, but our data supported that IS and IL-1β could induce apoptosis in cardiomyocytes, and this mechanism may account for cardiac dysfunction induced by AKI.

iPS cell technology enables the reprogramming of a wide variety of somatic cell types into various other types of cells. This technology offers a novel strategy for patient-specific derivation of clinically applicable lineage-specific cells, such as EPCs [15, 16, 49]. Directing the in vitro differentiation of iPS cells into functional EPCs may serve as a paradigm for human disease modeling and EPC-based therapies [15, 39, 40]. In the present study, iPS cells were differentiated into iEPCs, which expressed mature EPC markers such as CD133 and KDR. Thus, iPS cells can be used to derive numerous cells that can be differentiated into EPCs for transplantation purposes, as shown in this study. Moreover, recent evidence has revealed that iPS cells can also be induced to cells expressing markers of proximal tubules or podocytes [50–52]. Similar to the direct endothelial replacement by iEPCs in our study, Sharmin et al. demonstrated that the transplantation of human iPS cell-derived nephron progenitors, using spacers to release the tension of host kidney capsules, allowed the effective formation of glomeruli, in which iPS cell-derived podocytes accumulated around the fenestrated endothelial cells [52].

Tumorigenesis is a challenge in stem cell therapy. In some animal studies, direct implantation of iPS cells led to tumor formation [26, 53]. Therefore, directed differentiation into iEPCs should be conducted before transplantation to avoid the possibility of tumorigenesis. In this short-term study, we did not observe any abnormal cell proliferation after iEPC therapy. The long-term safety of iEPC therapy requires further exploration. Our data indicated that intravenous infusion of iEPCs did not cause abnormal proliferation in the heart or kidney (data not shown).

## Conclusions

The present report demonstrated the potential of human iPS cell-derived iEPCs as a novel therapy for ischemic AKI and remote cardiac dysfunction. The protective effects of this therapy most likely result from attenuation of apoptosis and inflammation. Further research is required to verify the long-term safety of this therapy.

## Additional file

Additional file 1: **Figure S1.** Characterization of human iPS cell-derived endothelial progenitor cells (iEPCs). Figure S2 Characterization of human iPS cell-derived endothelial progenitor cells (iEPCs). Figure S3 GFP-iEPC sorting. Representative flow cytometric analyses for (A) control iPS cells and (B) lentivirus-GFP-transduced iPS (GFP-iPS) cells. Figure S4 Gross appearance of kidneys with sham or AKI. Figure S5 Measurement of human angiogenesis-related proteins in the plasma of AKI mice. Figure S6 Increased plasma levels of brain natriuretic peptide in AKI patients. (DOC 7402 kb)

## Abbreviations

AKI: Acute kidney injury
Angpt: Angiopoietin
BNP: Brain natriuretic peptide
CO: Cardiac output
FGF: Fibroblast growth factor
I/R: Ischemia–reperfusion
IGFBP: Insulin-like growth factor-binding protein
LVEF: Left ventricular ejection fraction
LVFS: Left ventricular fractional shortening
MMP9: Matrix metallopeptidase 9
TGF-β1: Transforming growth factor-β1
VEGF-C: Vascular endothelial cell growth factor-C
