# Supplementary material for: Induced pluripotent stem cell-derived endothelial progenitor cells attenuate ischemic acute kidney injury and cardiac dysfunction
Source: Stem Cell Res Ther. 2018 Dec 10;9:344. doi: 10.1186/s13287-018-1092-x (PMC6288873; doi:10.1186/s13287-018-1092-x)
Supplement: Supplementary file 1 — Figure S1. Characterization of human iPS cell-derived endothelial progenitor cells (iEPCs). Figure S2 Characterization of human iPS cell-derived endothelial progenitor cells (iEPCs). Figure S3 GFP-iEPC sorting. Representative flow cytometric analyses for (A) control iPS cells and (B) lentivirus-GFP-transduced iPS (GFP-iPS) cells. Figure S4 Gross appearance of kidneys with sham or AKI. Figure S5 Measurement of human angiogenesis-related proteins in the plasma of AKI mice. Figure S6 Increased plasma levels of brain natriuretic peptide in AKI patients. (DOC 7402 kb) [file 13287_2018_1092_MOESM1_ESM.doc]

**Shen et al. Supplemental Material**

**Supplemental Methods**

**Approval for Human Studies**

This study was performed in compliance with the Declaration of Helsinki and was approved by the Institutional Review Board of National Taiwan University Hospital, Taipei, Taiwan (201111012RIB and 200907056R). All participants or their legal representatives provided written informed consent.

**Patients**

Consecutive patients were enrolled upon diagnosis with acute kidney injury (AKI). The definition of AKI was based on the criteria established by the Acute Kidney Injury Network.^1^ Briefly, AKI was defined as an absolute increase in serum creatinine of more than or equal to 0.3 mg/dL (≥26.4 μmol/L), an increase in serum creatinine of more than or equal to 50% (1.5-fold from baseline), or a reduction in urine output (documented oliguria of less than 0.5 mL/kg per hr for more than 6 hr) within 48 hr. To avoid the influence from other causes of chronic or severe heart dysfunction on the brain natriuretic peptide (BNP) level, the study excluded patients with chronic heart disease (based on chart reviews), with an abnormal echocardiogram on admission (i.e., systolic dysfunction: left ventricular ejection fraction [LVEF] < 55%; diastolic dysfunction: E wave/A wave ratio <0.75 or >1.5), acute coronary syndrome, acute myocardial infarction, acute pulmonary embolism, status post cardiopulmonary resuscitation, cor pulmonale, or respiratory failure with high positive end-expiratory pressure (>10 cmH_2_O). Plasma aliquots were frozen until analysis.

**Generation of Human Induced Pluripotent Stem Cells**

Skin fibroblasts from a 35-year-old married female patient with premature ovarian failure were used to generate induced pluripotent stem (iPS) cells after the patient’s informed consent was obtained.^2^ The skin fibroblasts were first expanded. Subsequently, lentivirus-expressing retroviral receptor Slc7a1 (Addgene, Cambridge, MA) and Virapower packaging mix (Invitrogen, Carlsbad, CA) were added to the dish plated with somatic cells (8 × 10^5^ cells/10-cm dish) in Dulbecco’s Modified Eagle’s Medium with 10% fetal bovine serum (FBS, Invitrogen). Slc7a1-expressing cells were selected using blasticidin (12 μg/mL). Plat-E packaging cells (8 × 10^6^ cells/dish, Cell Biolabs, Inc., San Diego, CA) with 1 μg/mL puromycin and 10 μg/mL blasticidin were transfected with 9 μg of pMXs-hOct4, pMXs-hSOX2, pMXs-hKlf4, and pMXs-hc-Myc (Addgene) using Fugene 6 (Roche, Mannheim, Germany). The Slc7a1-expressing somatic cells were then transfected with supernatant containing four types of retroviruses and polybrene (4 μg/mL, Sigma, St. Louis, MO). After 5 days, the transfected cells were replated onto dishes plated with mitomycin-C-inactivated mouse embryonic fibroblasts, and the culture medium was switched to primate embryonic stem cell medium supplemented with 10 ng/mL basic fibroblast growth factor (Invitrogen, Carlsbad, CA) after 24 hr. Human-iPS-cell-like colonies were manually selected 20 days after viral transduction. The culture medium was refreshed daily to maintain iPS cells in an undifferentiated state, and the cells were passaged once a week.

**Differentiation and Characterization of Human-iPS-Cell-derived EPCs**

To direct the differentiation of iPS cells into endothelial progenitor cells (iEPCs), half of the iPS cells were maintained in Endothelial Cell Growth Medium (EGM-2) comprising EBM-2 Basal Medium supplemented with SingleQuots Kit Supplements, growth factors (Lonza Inc., Allendale, NJ), and 20% FBS (Invitrogen). The medium was changed every other day for 7 days, after which the cells were used for in vitro and animal studies. For immunocytofluorescency, the cells were fixed with 4% paraformaldehyde (Bionovas, Ontario, Canada) for 30 min at room temperature (RT). They were then blocked with 10% normal goat serum in phosphate-buffered saline at RT for 1 hr and incubated at 4°C overnight in a 1:50 dilution of mouse antibodies against human CD31, kinase insert domain receptor (KDR), CD133, or VE-cadherin (BioLegend, San Diego, CA). Bound antibodies were detected by incubation with a 1:500 dilution of fluorescein isothiocyanate (FITC)-conjugated goat anti-mouse IgG antibodies or goat anti-rabbit IgG antibodies (Invitrogen) at RT for 1 hr. For flow cytometry, 5 ×10^6^ cells were incubated at 37°C for 1 hr in 150 μL of buffer with CD31, KDR, CD133, or VE-cadherin antibodies, followed by incubation with fluorescence-labeled secondary antibodies at RT for 1 hr. Uptake of 1,1’-dioctadecyl 3,3,3’,3’-tetramethylindo-carbocyanine (DiI)-labeled acetylated low-density lipoprotein (acLDL) is commonly used as a marker for the identification of EC. To verify that exposure to EGM-2 resulted in the differentiation to an endothelial cell-like phenotype, a fluorescence microscope was used to examine uptake of acLDL (Invitrogen) after incubation at 37°C for 4 hr. An in vitro tube formation assay was performed using an In Vitro Angiogenesis Assay Kit (Chemicon, Temecula, CA).^3^ Briefly, ECMatrix gel solution was thawed at 4°C overnight, mixed with ECMatrix diluent buffer, and placed in a 96-well plate at 37°C for 1 hr to allow the matrix solution to solidify. iEPCs were harvested and then placed in a matrix solution with EBM-2, in which the cells were incubated at 37°C for 16 hr. Tubule formation was inspected under an inverted light microscope. The tube number was calculated in three independent experiments using ImageJ software.

**Culture of HL-1 Cardiomyocytes**

HL-1 cells were maintained in Claycomb medium supplemented with 1 μM retinoic acid, 10 μM norepinephrine (Sigma-Aldrich), 100 units/mL penicillin, 100 μg/mL streptomycin, and an additional 1× nonessential amino acids (Life Technologies, Carslbad, CA). The medium was changed approximately every 24 hr. The cells were grown at 37°C in an atmosphere of 5% CO_2_ and 95% air at a relative humidity of approximately 95%. To study the effect of indoxyl sulfate (IS) and interleukin-1β (IL-1β) on cell apoptosis, HL-1 cardiomyocytes were incubated with 0.2 mM IS for 24 hr, followed by incubation with or without 0.2 ng/mL IL-1β for 4 hr in the continued presence of IS.

**Tissue Preparation for Pathological Examinations**

The mice were sacrificed after intraperitoneal injection of ketamine/xylazine (100/10 mg/kg, intraperitoneally) on day 2 after I/R surgery. The kidney and heart were carefully excised, fixed overnight at 4°C in 4% paraformaldehyde, and paraffin-embedded for obtaining tissue sections (5 μm).^4^

**Scoring of Tubular Injury**

Tubular injury was studied on renal sections stained with hematoxylin and eosin (Sigma-Aldrich). An investigator blinded to the samples scored the tubular injuries. In brief, images were captured using digital imaging (×200) sequentially over the entire sagittal section encompassing cortex and outer medulla (10–20 images). Each image was divided into 252 squares by a grid. The presence of tubule injuries, including tubule flattening, necrosis, apoptosis, or intratubular casts, resulted in a positive score. The final score was calculated based on the proportion of squares with positive scores in each image, which was averaged for all images from the individual kidney for each group.

**Evaluation of Microvessels**

Renal sections were incubated for 16 hr at 4°C with an antibody against mouse CD31 (1:100, Abcam, Cambridge, UK) and then incubated for 60 min at RT with a 1:400 dilution of goat anti-rat IgG antibodies, followed by development with 3,3′-diaminobenzidine (Sigma-Aldrich). Images were captured by digital imaging (×200) sequentially over the entire sagittal section encompassing cortex and outer medulla (10–20 images), according to the method modified from a previous publication.^5^ After excluding large vessels with hyalinized walls, all CD31+ microvessels were counted. A histogram was used to represent the quantitative evaluation of microvascular density (number per high power field, HPF). The final number of microvessels/HPF was the average for all images from the individual kidney for each group.

**Identification of Apoptosis Using TUNEL Staining**

Terminal dUTP nick-end labeling (TUNEL) staining was performed according to the manufacturer’s protocol (In Situ Cell Death Detection Kit, Roche). The sections were counterstained with 4’,6-diamidino-2-phenylindole (DAPI), and images were captured using digital imaging (×400). Apoptotic nuclei were identified using green fluorescence, and the numbers of TUNEL positive nuclei were averaged from five nonoverlapping regions of each tissue section in a high power field for each group.

**Cell Lysate Preparation and Western Blot Analysis**

To prepare cell lysates, the heart or cells were lysed for 1 hr at 4°C in 20 mM Tris-HCl, 150 mM NaCl, 1mM EDTA, 1 mM EGTA, 1% Triton X-100, and 1 mM PMSF at pH 7.4 with protease inhibitors (Cell Signaling Technology, Danvers, MA). The lysates were then centrifuged at 11800 ×g for 30 min at 4°C, and the supernatant was collected using a previously described method.^6^ The protein concentration of the supernatant was measured. An aliquot of the supernatant (20 mg/mL protein) was subjected to 8%–12% SDS-PAGE, and the proteins were transferred onto polyvinylidene difluoride membranes (Millipore, Darmstadt, Germany). The membranes were incubated overnight at 4°C with a 1:1,000 dilution of polyclonal antibodies against Bax, Bcl-2 (GeneTex, Irvine, CA), or caspase 3 (Abcam) in TBST. Subsequently, the membranes were incubated for 1 hr at RT with horseradish peroxidase (HRP)-conjugated goat anti-rabbit IgG antibodies (1:5,000 in TBST; Sigma-Aldrich), and bound antibody was detected using Chemiluminescence Reagent (NEN Life Science, Boston, MA). The intensity of each band was quantified using a densitometer. Glyceraldehyde 3-phosphate dehydrogenase (GAPDH), used as the internal control, was detected using rabbit anti-GAPDH antibody (1:20000 dilution in TBST; GeneTex) and HRP-conjugated goat anti-rabbit IgG antibody (1:5000 dilution in TBST; GeneTex). Bands were identified using an ECL detection kit. Relative protein expression levels were quantified through Western blotting with UN-SCAN-IT gel 6.1 software (Silk Scientific, Inc. Orem, UT) after densitometric scanning of the exposed films.

**Evaluation of Renal Function**

Plasma creatinine and blood urea nitrogen levels were measured in the Department of Laboratory Medicine, National Taiwan University Hospital.

**Measurement of Plasma Interleukin-1β**

Plasma IL-1β was measured using commercially available enzyme-linked immunosorbent assay (ELISA) kits (mouse and human kits from RayBiotech, Norcross, GA and Abcam, respectively) according to the manufacturer's instructions.

**Measurement of Plasma Brain Natriuretic Peptide**

Human plasma BNP was measured using Abbott's AxSYM automated analyzer (Abbott Laboratories, Abbott Park, IL). Mouse plasma BNP was measured using a commercially available ELISA kit (RayBiotech) according to the manufacturer's instructions.

**Measurement of Plasma Indoxyl Sulfate**

In a water bath at RT, 20 μL of the plasma was mixed with 80 μL of acetonitrile (J. T. Baker, Phillipsburg, NJ) in a 1.5-mL microcentrifuge tube, followed by vortex mixing for 60 s. The tube was then centrifuged at 1860 ×g for 10 min, and the supernatant was transferred to a sample vial. A 10-μL aliquot of the supernatant was injected into a high performance liquid chromatography (HPLC) system. HPLC was performed using a Waters 600 Solvent Delivery Pump (Waters, Milford, MA, USA) equipped with a fluorescence detector (Surveyor FL plus detector, Thermo Fisher Scientific, MA, USA). A reversed-phase C18 column (SHISHEDO CAPCELL PAK type SG C18, 5 μm, 150 mm × 4.6 mm) was used, and the mobile phase was composed of acetonitrile/water (8:92, v/v) containing 0.2% trifluoroacetic acid (Alfa Aesar, Ward Hill, MA). Isocratic elution was applied, and the mobile phase was delivered at flow rate of 1.0 mL/min. The excitation and emission wavelengths of the fluorescence detector were set at 280 and 375 nm, respectively. Calibration samples of IS were prepared at 1.7, 3.9, 8.8, 9.8, 44.4, and 100 μM in plasma. The calibration curve was calculated with the peak area against the spiked concentrations. IS was then determined according to the measured peak area.

**Measurement of Circulating Human Cytokines**

According to the manufacturer's instructions, a Human Angiogenesis Array Kit (R&D Systems, Minneapolis, MN) was used to analyze human angiogenesis-related proteins in plasmas collected from AKI mice on day 2 after vehicle or iEPC therapy. Briefly, 100 μL of plasma was mixed with a cocktail of biotinylated detection antibodies. The mixture was then incubated overnight at 4°C with a nitrocellulose membrane spotted with capture antibodies in duplicate. Protein-detection antibodies bound to the capture antibodies were detected using streptavidin–HRP and chemiluminescent detection reagents. Mean spot pixel densities representing protein expression were analyzed using UN-SCAN-IT gel 6.1 software (Silk Scientific, Inc.). The pixel densities of angiogenesis-related proteins relative to those of reference spots were expressed.

**Echocardiographic Analysis**

Mice were lightly anesthetized with isoflurane (30 mg/kg). M-mode echocardiograms were obtained from the short axis view of the left ventricle at the tip of mitral valve leaflets using a prospect, ultrasound machine (S-Sharp, New Taipei City, Taiwan). Measurements included left ventricular fractional shortening, left ventricular ejection fraction, stroke volume, and cardiac output.

**References**

**Supplemental Figures**

**Figure S1.** Characterization of human iPS cell-derived endothelial progenitor cells (iEPCs). (A, B) Bright-field images depicting colony formation of iPS cells and iEPCs. Scale bar = 500 μm. (C) iPS cell colonies with embryoid bodies were observed. Scale bar = 500 μm. (D) Bright-field images depicting the cobble-stone-like morphology of the iEPCs. Scale bar = 500 μm. (E) iPS cells did not uptake acetylated-LDL. Scale bar = 500 μm. (F) Differentiation into iEPCs was demonstrated using acetylated-LDL incorporation. Scale bar = 50 μm. (G, H) Bright-field images depicting the vascular tube formation of iEPCs cultured on Matrigel. iPS cells did not form vasculartubes on Matrigel. iPS cell colonies were observed with embryoid bodies. Scale bar = 5 um.


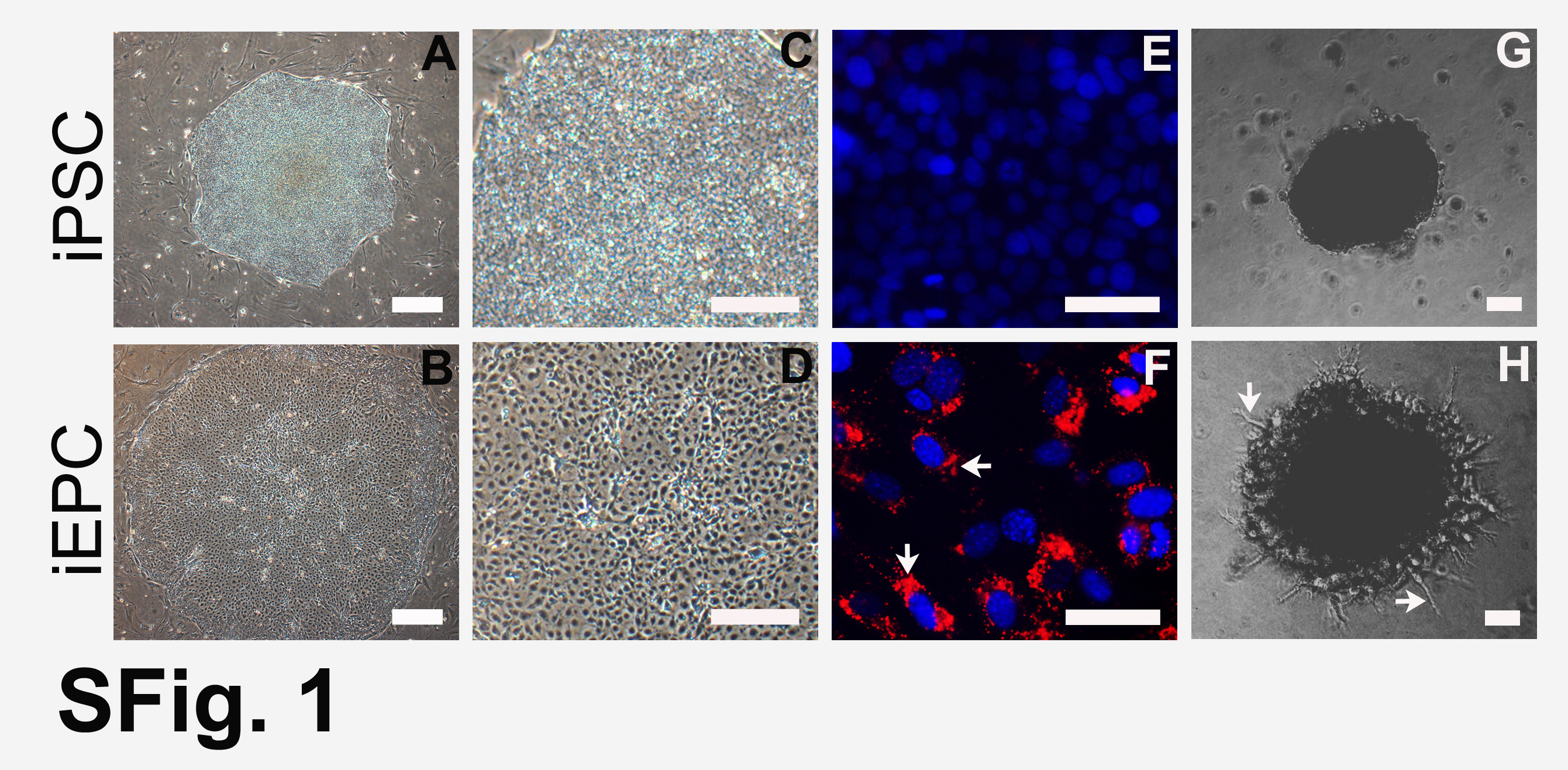


**
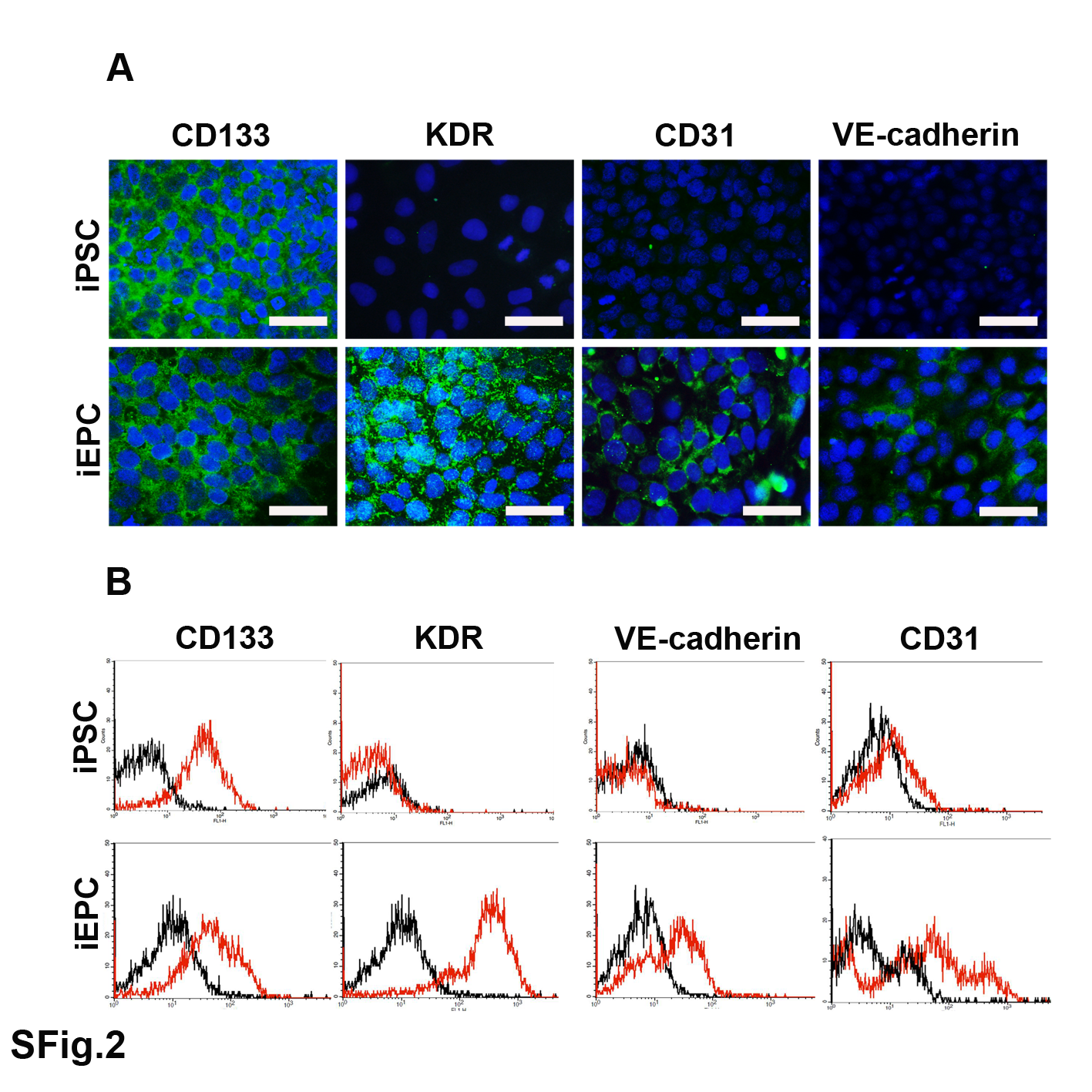
**

**Figure S2.** Characterization of human iPS cell-derived endothelial progenitor cells (iEPCs). (A) Representative fluorescence images illustrating CD133, KDR, CD31, and VE-cadherin staining. Scale bars = 50 μm. (B) Representative flow cytometric analyses for the expression of CD133, KDR, CD31, and VE-cadherin.

**
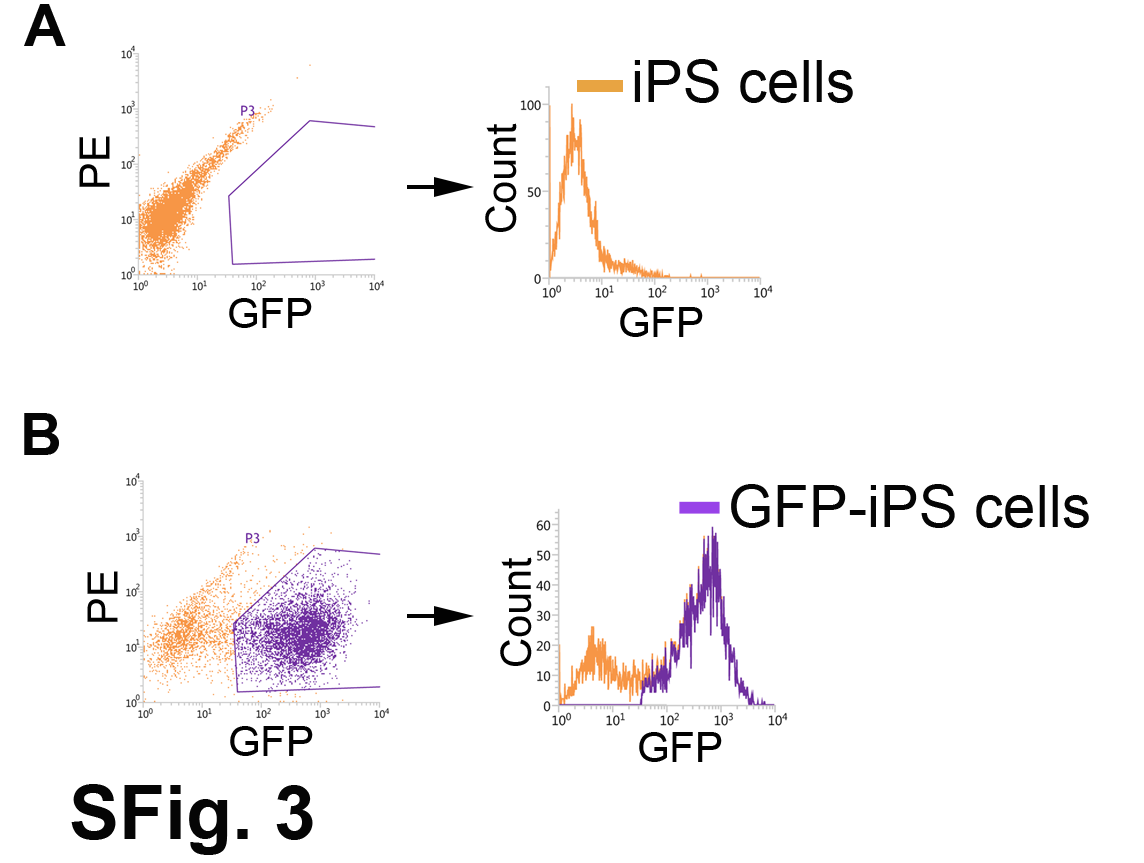
**

**Figure S3.** GFP-iEPC sorting. Representative flow cytometric analyses for (A) control iPS cells and (B) lentivirus-GFP-transduced iPS (GFP-iPS) cells.

**
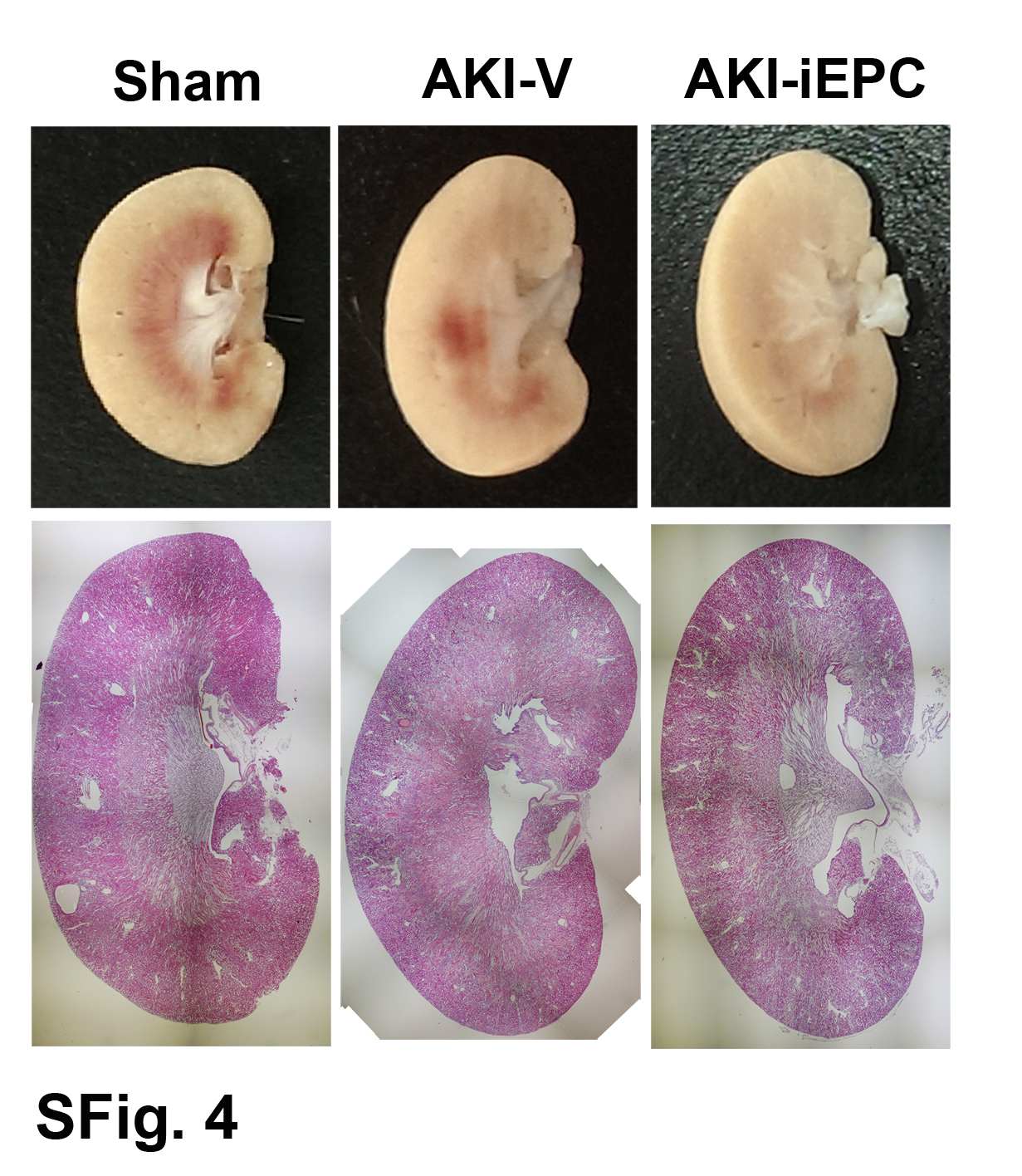
**

**Figure S4.** Gross appearance of kidneys with sham or AKI. (A) Representative gross appearance of kidneys (upper panel) and hematoxylin and eosin-stained renal sections (lower panel) in mice on day 2 after sham surgery or renal ischemia/reperfusion injury (AKI) with vehicle (AKI-V) or systemic iEPC therapy (AKI-iEPC).

**
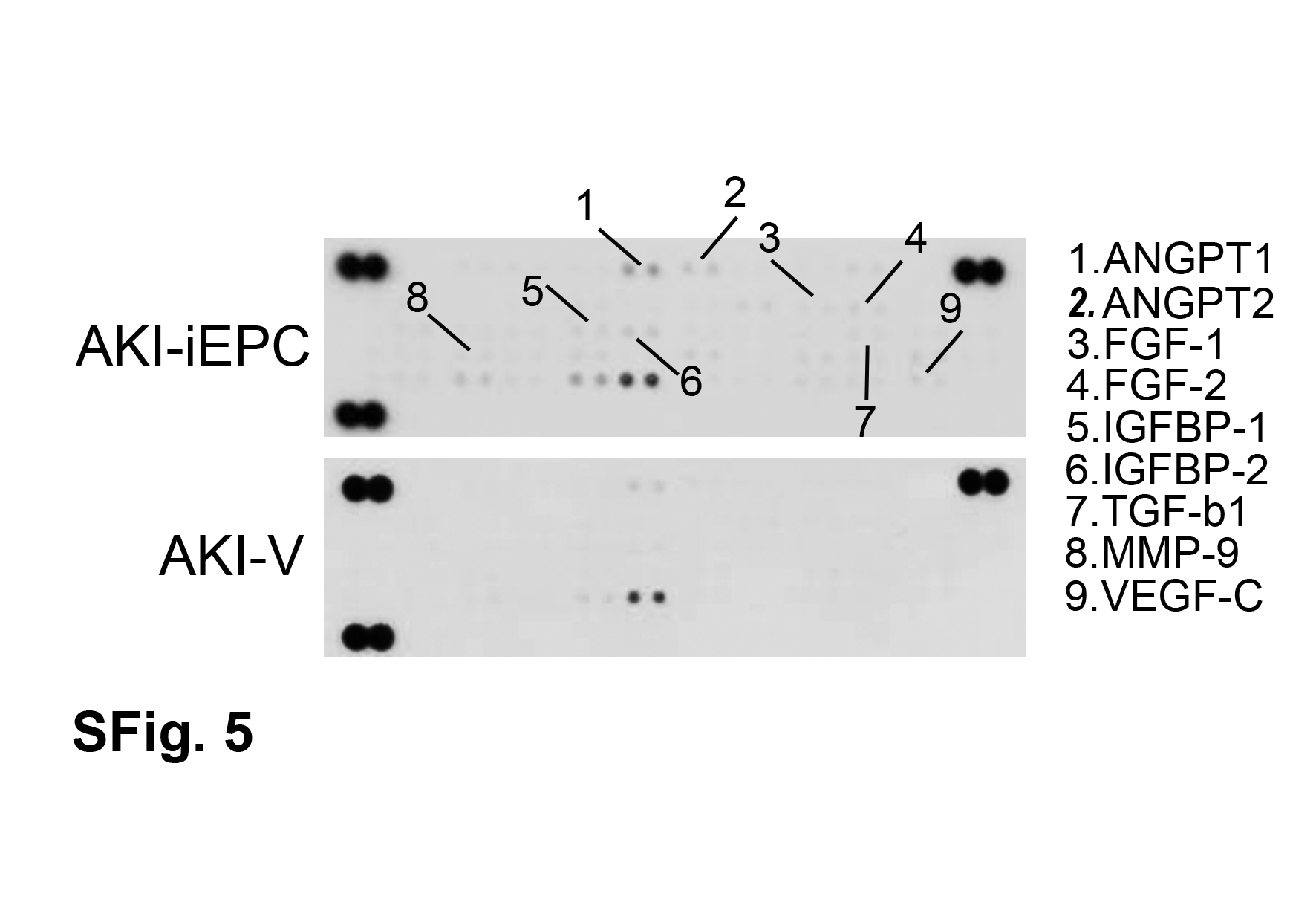
**

**Figure S5.** Measurement of human angiogenesis-related proteins in the plasma of AKI mice. Representative arrays from the detection of human angiogenesis-related proteins in the plasma of AKI mice using Human Angiogenesis Array Kit (R&D Systems).

**
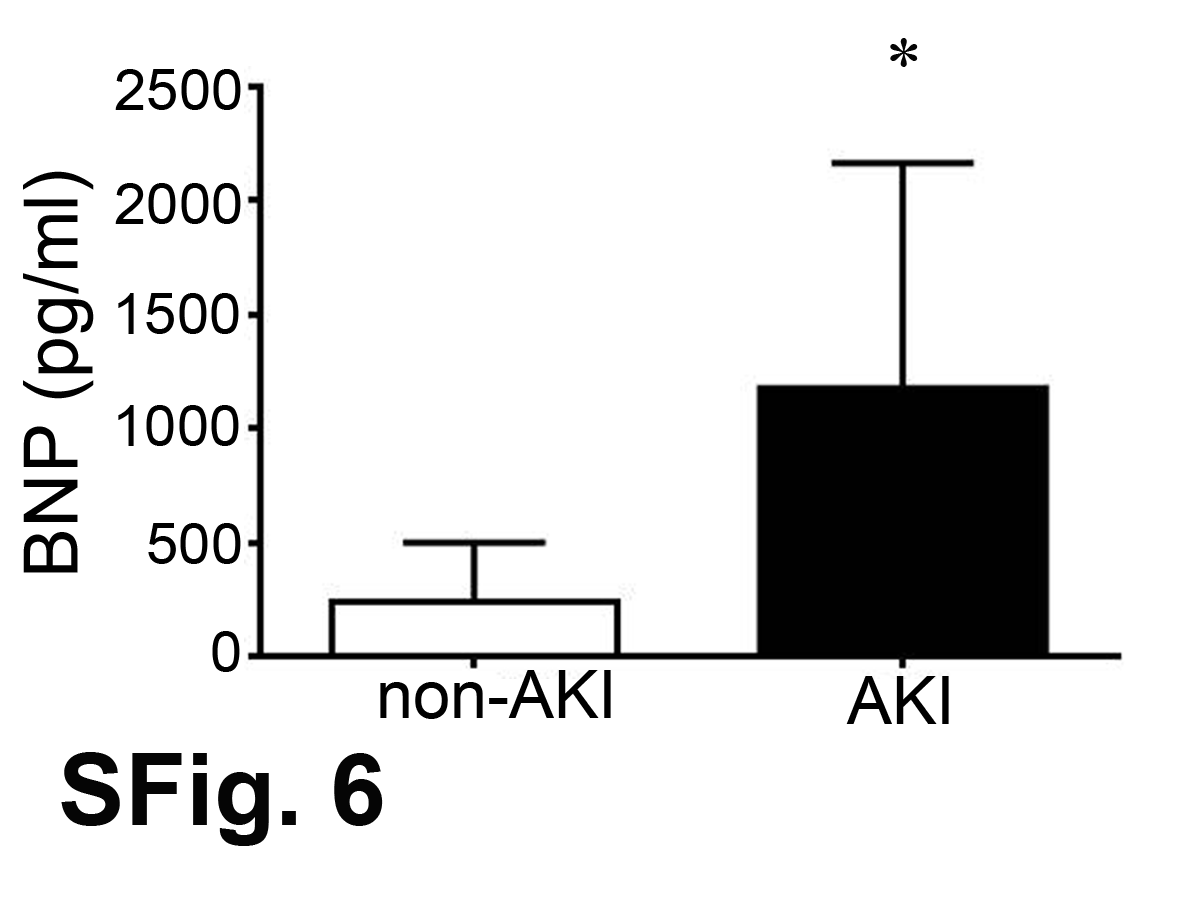
**

**Figure S6.** Increased plasma levels of brain natriuretic peptide in AKI patients. Bar chart indicating plasma levels of brain natriuretic peptide (BNP) in AKI and non-AKI patients on day 2 after diagnosis. Data are expressed as mean ± SEM. N = 25 per group. **P* < 0.05 vs. non-AKI group.
